# Dialect culture and the utilization of public health service by rural migrants: Insights from China

**DOI:** 10.3389/fpubh.2022.985343

**Published:** 2022-11-10

**Authors:** Qingjun Zhao, Siyu Xu, Noshaba Aziz, Jun He, Yue Wang

**Affiliations:** ^1^College of Economics and Management, Nanjing Agricultural University, Nanjing, China; ^2^School of Economics, Shandong University of Technology, Zibo, China; ^3^Institute of Agricultural Economics and Development, Jiangsu Academy of Agricultural Sciences, Nanjing, China

**Keywords:** public health services, dialect culture, rural-to-urban migration, China, cultural barriers

## Abstract

The right to health is a fundamental human right for human beings to live in dignity. Everyone has the right to enjoy the fair and accessible highest standard of health by utilizing public health services. However, access to essential public health services also highly depends on the dialect culture. It is believed that the dialect culture also influences the efficiency of public health policies. To explore the phenomenon empirically, the current study utilized data sourced from geographical distribution information of Chinese dialects and the China Migrants Dynamic Survey for 2017. The study employed the Probit, IVprobit, and Eprobit models to estimate the impact of dialect culture on migrants' use of public health services. The findings revealed that the dialect culture significantly hinders the migrants' utilization of public health services. Further, by employing heterogeneity analysis, the findings revealed that the results are more pronounced in migrants, born after 1980, and are female with low educational background and also those migrants having local medical experiences and moving toward non-provincial cities. Finally to explore the mechanism of dialect culture influencing migrants' public health service, the study employed mediation analysis and KHB Method. The findings revealed that information transmission, health habits, social capital, and cultural identity are the potential pathways influencing the migrants' use of public health services. The findings conclude that rural-to-urban migrants' access to public health services is influenced by their cultural adaptation. Hence, the study proposes that the government should amend the policy inefficiency concerns caused by cultural differences and strengthen the regional cultural exchanges to build trust.

## Introduction

Health is an inclusive state of mental, physical, and social wellbeing. According to the Constitution of the World Health Organization (1), attaining the highest health standard is considered a fundamental human right for every individual. Health is human capital and is considered as durable as physical capital (2). But health depreciates with time; that can be maintained with improved health systems. Public health system also plays vital role in guarding against uncertain catastrophes such as COVID-19 pandemic. So providing equity in health care access is regarded as the main factor by Universal Declaration of Human Rights organization (3). There are many factors that lead to inequalities in availing health services utilization (4, 5). For instance, low income, poor living conditions, and reduced access to health services lead people to the worst health, especially impoverished populations who often access fewer health services despite higher demand. Moreover, in the context of rural-urban migration; various factors influence the health services of the migrant people. Migrant workers are less likely to establish health records, participate in health education programmes, and seek medical care. So the point of interest is how to expand the provision of health services for most rural populations. In this study, we emphasized China; China is the largest developing country, which witnesses millions of migrations of rural people annually from rural to urban areas. According to the “Statistical Bulletin of the People's Republic of China on National Economic and Social Development in 2021” (National Bureau of Statistics of China), the total number of rural migrants in China reached 292.51 million in 2021, which accounted for 20.71% of the country's total population. Moreover, it is also stated that comparatively, urban residents and rural migrants are more vulnerable to infectious diseases. Insufficient supply, accompanied by low service quality, makes migrants' health even worse. In this regard, the utilization of public health services by rural migrants is regarded as a crucial phenomenon to be explored. It is generally believed that rural migrants cannot enjoy the same public services and social benefits as registered inhabitants especially basic public health services (6, 7).

In 2009, China launched the National Health and Family Planning Commission and emphasized equalizing essential public health services for migrant people. Likewise, the 19th National Congress of the Communist Party of China also emphasized accelerating the equalization of essential public services. Further, the “14th Five-Year Plan for National Economic and Social Development of the People's Republic of China and the Outline of Vision 2035” in 2020 also aimed at strengthening the public health system. Due to continuous efforts, the health policies at the national level in China improved; per capita, the primary public health service funding standard in China rose from 17 yuan in 2009 to 79 yuan in 2021. The services also increased from the initial nine categories to 14 categories. Despite the positive repercussion, people's awareness and utilization of essential public health services are not improved, especially among rural migrants (8, 9). That led to the waste of medical and health resources and also influenced the supply of essential public health services effectiveness. The previous literature revealed that place of residence is associated with disparities in health services (10–12). Moreover, China is a multi-ethnic and multi-lingual country with a vast territory, a large population, and a long history. Different regions have formed rich and complex regional cultures. The cross-regional flow of rural migrants alters the cultural environment, influencing their utilization of public health services. In the healthcare sector, it is believed that culture and linguistic incompetence significantly influences healthcare delivery, healthcare consumption, and health outcomes (13). It is evident in various communities and a region, including China, that multilingualism (the presence of more than one language) adversely affects health (14). In this regard, Zhang et al. (15) proposed opting for interventions such as professional interpreter service, service-learning interpreter programs, or mobile interpreting apps that are medically accurate and culturally sensitive for dialectally diverse China.

In the prevailing literature, many researchers explored migrants' use of public health services by focusing on factors such as age, education level, marital status, family income, residence time, migration scope, social network, medical insurance, health status, and air pollution (16–20). However, regarding demanders for public health services, rural migrants are likely to have a relatively recessive factor derived from their characteristics, resulting in a shortage of public health services at the individual level. Moreover the dialect culture is also likely to influence the public health services primarily in the context of migrants, as patients with linguistic barriers are subject to unnecessary health services, undesirable outcomes, and excess healthcare costs (13). These communication barriers in healthcare institutions without formal language support may have an undesirable impact on healthcare delivery and the patient-healthcare provider relationship. So based on the above discussion, the current study aims to explore the influence of dialect culture on the utilization of public health services by rural migrants. The study further explores the mechanism between dialect culture and the utilization of public health services. As per the authors' knowledge, these notions remained under-researched in the existing literature generally and especially in the context of China.

The rest of this paper is structured as follows: the next section Literature review and hypothesis development reviews the literature on dialect culture and rural migrants' utilization of public health services and puts forward the research hypothesis. Section Methodology introduces the primary variables, data sources, and estimation strategies. The empirical results are presented in section Empirical results. Section Discussions discusses the main findings of this study and compares them with existing research. In last, section Conclusion and policy implications concludes the study with several policy implications. The study limitations are also presented in this section.

## Literature review and hypothesis development

This section attempts to sort out and review recent literature on the relationship between rural migrants' public health services utilization and language research. Firstly, the relevant research regarding the factors of the utilization of public health services for rural migrants is explored. Then the impact of language on the public health service of rural migrants is revealed. Based on the literature, the current study proposed the hypothesis and conceptualized the framework operationalized.

Concerning the utilization rate of public health services for rural migrants, the existing studies have explored the phenomenon based on three aspects: the formulation of public health service policies, the implementation of public health service policies, and the demanders themselves. From the formulation of public health service policies, public health service costs influence the rural migrants' public health services utilization and the population they cover. Some studies revealed that high cost increases treatment and prevention services utilization (21, 22). In contrast, some studies revealed that higher cost reduces public health service coverage (23–25). In the context of medical insurance, it is found that it can significantly improve the utilization of public health services for rural migrants (26, 27). The study by Hong et al. (28) and Chen et al. (29) revealed that the lack of medical insurance rights and interests of rural migrants often causes them to be unable to seek medical treatment due to illness, and minor illnesses lead them to significant distress and even lead them to lose their ability to work. From the perspective of implementing public health service policies, Li et al. (30), in his study, revealed that the government departments do not adequately publicize essential public health services, which leads to the lack of understanding of the policies by the rural migrants. As a result, public health services are not effectively utilized (31). The study of Suphanchaimat (32) found that interactions between healthcare providers and migrant patients also influence the migrants' public health services utilization. For individual factors, Chiu (33) argued that the prevalence of mental health factors and the use of mental health services vary widely across ethnic groups. Likewise, Celik (34), Ahmed (35), and Tzogiou (36) revealed that socioeconomic factors of migrants, such as occupational category, living conditions, and income status, also lead to the unequal utilization of public health services by migrants. Furthermore, social integration and discrimination also severely undermine the cross-border healthcare utilization among migrants of Russian descent (37). It is also believed that higher education levels are associated with higher levels of migrant acceptance of new medicines. Several studies affirmed that educational levels significantly increase women's utilization of public services (38–42).

Some studies also analyzed the impact of language on the utilization of public health services for rural migrants. The study of Peled (43) revealed that the globalization and normalization of population mobility have become increasingly prominent; as a result, the issue of crossing language barriers has brought significant challenges concerning the quality and equitable provision of health services. Likewise, Lara (44) revealed that migrants positively correlate the degree of cultural adaptation and utilization of medical resources. Communication barriers mainly cause the low utilization rate of medical resources. A recent study by Khatri and Assefa (45) also revealed that language and communication problems adversely influence people's access to public health services. Likewise, Wang et al. (46) also found that language and culture influence the public health services accessibility to older migrants in the case of Canadian migrant workers. Another study by Salami et al. (47) also exhibited the same findings and unveiled that language is the main barrier to accessing and utilizing mental health services by migrants. Compared with Canadian and American migrants in English, the utilization rate of limited English immigrant public health services is significantly higher (48). Rasi (49) also revealed that cultural and language differences unfavorably influence the communication between migrant patients and public health professionals, hindering the migrant patients' accessibility to health care services.

Based on the literature, it is found that only a handful of studies explored the relationship between language barriers and public health service utilization by international migrants. In the case of rural domestic migrants, the studies are also found scarce. The main difference between rural migrants and residents is that they were born and raised in different places. In a specific growth environment, rural migrants have long been deeply branded with the imprint of regional culture. Behind the transfer from rural to urban areas are cultural conflicts and adaption between different regions. Lu et al. (50) analyzed the impact of language barriers on the health status of the elderly migrants. They found that language barriers reduced the ability of the elderly migrants to build social networks, which led to poorer health status.

**Figure 1:**
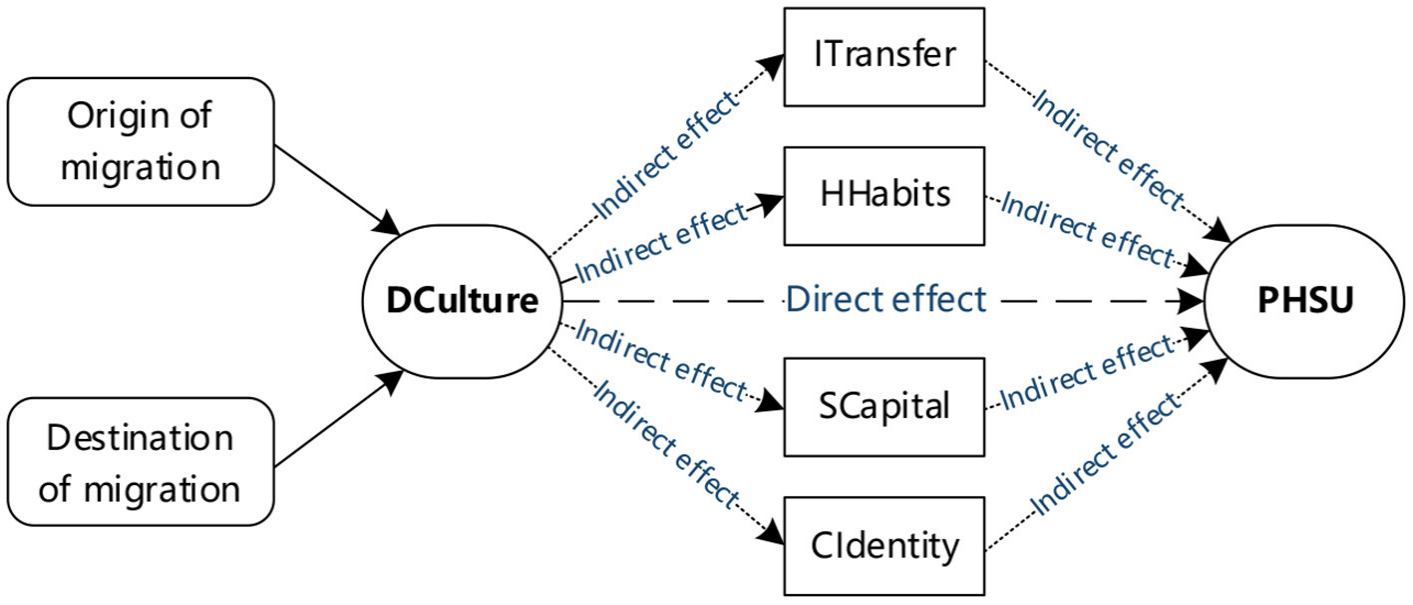
Framework operationalized in the current study.

Further, as a typical informal system, regional culture has a subtle and profound impact on the cognition, interaction, and strategic choice of rural migrants in the process of using public health services. Many features may contribute to this phenomenon; firstly, having similar cultural backgrounds induces communication and coordination between two groups, while cultural differences confront challenges (51). Moreover, the greater the dialect cultural differences, the higher the communication cost between rural migrants and public health service providers, and the greater the barriers to information transmission. Thus, it reduces the utilization rate of public health services. Secondly, the more significant the dialect cultural difference between the source and destination of rural migrants, the greater the difficulty in the cultural adaptation of rural migrants, and the lower the degree of convergence with residents' health habits and lifestyles (52). So, it will create barriers and hinder the utilization of public health services. Thirdly, due to the impact of regional culture, rural migrants who have left their hometowns are more inclined to interact with their villager fellows having similar cultures, which results in the lack of ways to extend outwards and the inability to establish and expand social networks and reconstruct social capital in the influx of cities. That further makes it difficult to obtain accurate information on local public health service projects and ultimately results in the low utilization rate of public health services. Finally, it is difficult for individuals with different regional and cultural backgrounds to establish a trusting relationship and a sense of cultural identity due to differences in their way of thinking, value orientation, and behavioral norms. Cross-cultural mobility is likely leading to a gap of trust between rural migrants and public health services, so rural migrant workers are inactive to respond to the public health service's management, and health education inhibits them from using public health services. Thus, this paper constructs the analytical framework shown in Figure 1 and proposes the following research hypotheses:

H1: Dialect culture (“DCulture”) significantly hinders the utilization of public health services (“PHSU”) for rural migrants.
H2: DCulture indirectly hinders rural migrants' access to PHSU through information transfer (“ITransfer”), healthy habits (“Hhabits”), social capital (“SCapital”), and cultural identity (“CIdentity”).

## Methodology

### Data sources

This study used data from China Migrants Dynamic Survey (“CMDS”) for 2017. This survey was conducted by the National Health and Family Planning Commission of China, which is authoritative and time-sensitive. The survey adopted the stratified, multi-stage, and probability proportionate to size sampling method. The survey covers the inflow places where the floating population is relatively concentrated among mainland China's 31 provincial-level administrative units. The data is collected from people aged 15 and above without household registration in the district (county, city). The survey content involves the basic information of the floating population and their family members, the scope and trend of migration, the utilization of essential public health services, and the management status of marriage, childbirth and family planning services, etc., which has both professional, scientific and large-scale characteristics, and can comprehensively describe the utilization of public health services of rural migrants in China. The total number of CMDS2017 data samples is 169,989. Since this study focuses on the rural immigrant group and only the floating population who has lived locally for more than 6 months and used essential public health services, the valid screened samples found are around 117,108.

### Study variables

#### Explained variables

The explained variable in the current study is “utilization of public health services.” “Documentation of health records” is employed as the indicator reflecting the utilization of public health services of rural migrants. The reason is that the current territorial governments mainly rely on health records to carry out health education, prevention, and control of infectious diseases for the floating population and pregnant women. In the questionnaire, this indicator was operationalized as “whether a resident health file has been established for you,” and the respondents answered “yes, it has been established,” “not established, never heard of it,” “not established, but heard of it” and “Unclear.” This paper assigns “Yes, has been established” to 1, and other cases are assigned to 0. According to the data, it is found that around 33,634 rural migrants have established health records within the sample, accounting for 28.72%.

#### Explanatory variable

**Figure 2:**
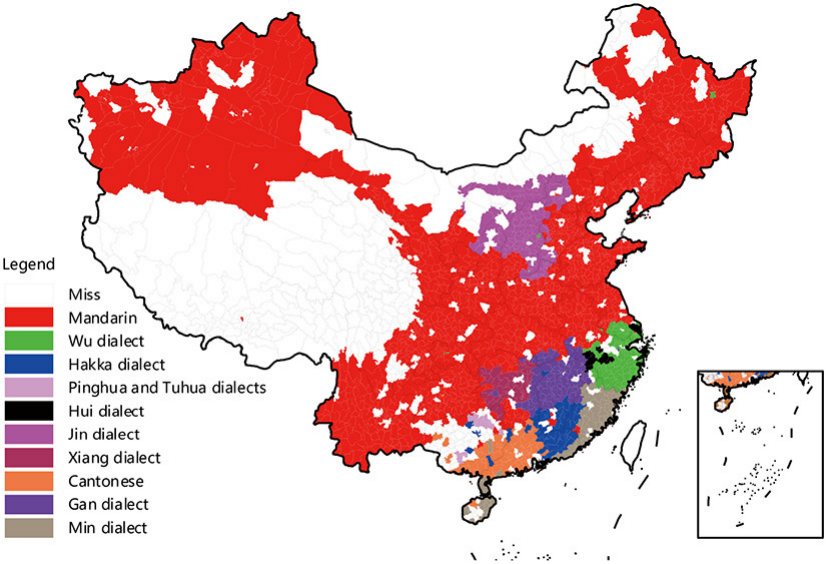
The geographic distribution of the ten major dialect regions in China [Data source: The Chinese Dialect Dictionary (Revised Edition)].

The introduction explains that China has a vast territory, substantial cultural differences across regions, and diverse Chinese dialects. Rural migrants face enormous language challenges in adapting to life in their places of origin. Although Mandarin is regarded as a formal and official language of China daily, rural migrants mostly use the Chinese dialect as the mode of communication and social interaction (50). At the same time, language is a comprehensive representation of culture, which can account for culture's inheritance, differentiation, and assimilation. Therefore, based on the current research results and the particular national conditions of China's multilingualism, this study constructed dialect cultural indicators based on the geographical distribution of dialects. According to the Chinese dialect division in the 2020 “Chinese Dialect Dictionary (Revised Version),” the Chinese dialect level in China is divided into three levels: dialect region, dialect area, and dialect piece. Accordingly, there are ten major dialect regions, 17 dialect areas, and 97 dialect pieces, covering 2,596 counties (cities, districts, and flags). Figure 2 shows the geographic distribution of the 10 Chinese dialect region. As shown in Figure 2, Chinese dialect areas in China vary greatly from provincial boundaries; a province may have multiple dialect areas, and a dialect area may also span multiple provinces. Overall, Mandarin is the most spoken Chinese dialect.

Although the unit of dialect is different from the administrative unit, there is often only one dialect piece in a county. Drawing on the research of Liu et al. (53), this paper first uses 97 dialect pieces to identify the language types of the counties in the origin and inflow of rural migrants. It then measures the dialects and cultural differences between the origin and inflow of rural migrants based on the dialect distance between the counties. The specific rules are as follows: when two counties belong to the same dialect piece, the dialect distance between the counties is 1; when they belong to different dialect pieces in the same dialect area, the dialect distance is 2; when they belong to different dialect area in the same larger dialect area, the dialect distance is 3; when they belong to different larger dialect area, the dialect distance is 4. The larger the value, the more significant is the dialectal cultural difference between the source and inflow areas of rural migrants. Among the rural migrants, it is found that about 31.01% of the same dialect moved, 17.43% of different dialects piece in the same dialect area, 17.99% of different dialects area in the same larger dialect area, and 33.57% of different larger dialect area.

**Figure 3:**
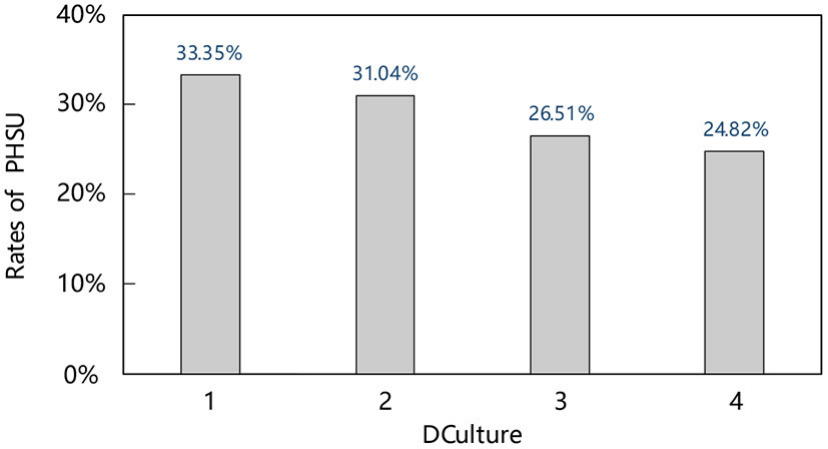
Rates of PHSU among rural migrants in different DCulture.

Figure 3 further intuitively presents the utilization of public health services for rural migrants under different dialect and cultural differences. According to the figure, it is shown that the more significant the dialect cultural difference, the lower the utilization rate of public health services for rural migrants. Specifically, 33.35% of rural migrants in the same dialect area have established health records, 31.04% in the same dialect area with different dialect pieces, 26.51% in the same dialect region with different dialect areas, and the proportion of rural migrants flowing in different dialect region was only 24.82%. This shows that dialect culture hinders the utilization of public health services for rural migrants, but the causal relationship needs further testing.

### Other variables

It is believed that dialect culture is an endogenous variable. So in this regard, the current study uses the absolute value of the difference between the topographic relief of the source of rural migrants and the topographical relief of the inflow area as an exogenous geographic instrument variable. The data comes from the Global Change Scientific Research Data Publishing System (www.geodoi.ac.cn). The reasons for using the difference in terrain relief as a geographic instrument variable are as follows: Generally speaking, the greater the degree of surface segmentation in a particular area, the higher the average elevation. And the greater the degrees of mountain relief, the more efficiently the area's residents are affected by such a complex geographical environment in China. And the isolation of the geographical environment leads to the relative independence of language communication and cultural development between different communities, which have evolved into cultural differences and dialect diversity between regions for a long time (53, 54). Therefore, the topographic relief difference and the dialect cultural variables should be positively correlated. Still, the topographic relief is a natural geographical condition formed in the region's history, which exists objectively and is not directly related to the utilization of public health services for rural migrants. Therefore, the terrain relief difference is regarded as a more appropriate instrument variable in the current study.

The paper further considers information transmission, health habits, social capital, and cultural identity to assess the mechanisms of dialect culture that influence the utilization of public health services for rural migrants. The information transmission is measured by the questionnaire item “Have you heard of the national basic public health service project.” The options to this question include “heard of” and “haven't heard of it, “which assign a value of 1 and 0, respectively. Within the sample, it was found that about 69,224 rural migrants had heard of the national basic public health service project, accounting for 59.11%. Further, health habits are measured by items including, “My hygiene habits are quite different from those of local citizens,” and the options include “completely agree,” “basically agree,” “disagree,” and “completely disagree.” “Disagree” and “Totally disagree” are combined and assigned a value of 1, and “completely agree” and “basically agree” are assigned a value of 0. Within the sample, about 93,459 rural migrants did not agree that their hygiene habits differed from local citizens, accounting for 79.81%. Furthermore, social capital is measured by “who do you interact with the most in your spare time (excluding customers and other relatives),” and the respondents answered the options, including “compatriots,” “other locals,” “other foreigners,” and “rarely with people.” This paper combines “other locals” and “other foreigners” and assigned a value of 1, and the rest of the cases are assigned a value of 0. Within the sample range, 49,009 rural migrants have the most exchanges with foreigners and locals in their spare time, accounting for 41.85%. Cultural identity is measured by “it is more important for me to do things according to the customs and habits of my hometown.” The options answered by the respondents include “completely agree,” “basically agree,” “disagree,” and “completely disagree.” “Totally Disagree” is merged with a value of 1, and “Totally Agree” and “Basically Agree” are assigned a value of 0. Within the sample, 49,772 rural migrants, or 42.50%, do not agree that it is vital to do things according to the customs of their hometown.

The study also controlled for various potential confounding factors that may simultaneously affect the utilization of rural migrants' public health services and migration decisions, mainly including gender, age, marital status, education level, self-assessment of health, migration time, range of migration, income level, medical distance, medical insurance, etc. In addition, considering the differences in the level of social and economic development in different cities, this paper matches the “China Urban Statistical Yearbook 2017” with the CMDS 2017 data and selects urban characteristics such as the level of medical services and city size in the inflow area as control variables. The definition of variables with their descriptive statistics is shown in Table 1.

**Table 1:** Descriptive statistics.

**Variables**	**Definition**	**Mean**	**Std. Dev**.	**Min**	**Max**
PHSU	Utilization of Public Health Service: Established = 1; Other = 0	0.287	0.452	0	1
DCulture	Dialect culture: same dialect piece = 1; different dialect piece in the same dialect area = 2; different dialect areas in the same dialect region = 3; different dialect region = 4	2.541	1.241	1	4
Gender	Male = 1; Female = 0	0.515	0.500	0	1
Age	Respondent's age in 2017 (years)	35.905	10.565	15	96
Marriage	Married = 1; Unmarried = 0	0.834	0.372	0	1
Education	High school and above = 1, below high school = 0	0.332	0.471	0	1
Shealth	Healthy or basic health = 1; unhealthy but able to take care of life or unable to take care of life = 0	0.973	0.162	0	1
Rtime	Duration in the host city (years)	6.299	5.982	0	64
FRange	Scope of mobility: inter-provincial mobility = 1; intra-provincial inter-city = 2; intra-city inter-county = 3	1.707	0.760	1	3
HIncome	Income level: logarithm of monthly per income household income of respondents	7.561	0.734	0	11.513
HDistance	Distance to hospital: within 15 mins = 1; 15–30 mins = 2; 30 mins−1 h = 3; more than 1 h = 4	1.179	0.441	1	4
Medicare	Medical Insurance: participated = 1; Not Participated = 0	0.194	0.395	0	1
PHospital	Medical service Level: per capita hospitals flowing into the city in 2016	0.579	0.570	0.104	8.929
SCity	City size: small and medium cities = 1; large cities = 2; mega cities = 3; more mega cities = 4	1.987	0.928	1	4
Instrument variable	The absolute value of the difference between the relief of the source and the inflow	0.448	0.777	0	5.784
ITransfer	Information transmission: heard of = 1; never heard of = 0	0.591	0.492	0	1
HHabits	Healthy habits: disagree or totally disagree = 1; totally agree or basic agree = 0	0.798	0.401	0	1
SCapital	Social capital: other natives or other outsiders = 1; other = 0	0.418	0.493	0	1
CIdentity	Cultural identity: disagree or totally disagree = 1; totally agree or basic agree = 0	0.425	0.494	0	1

### Model specification

#### Main effects model

Since the explained variable of the utilization of public health service in this paper is a discrete binary variable, so the probit model is used for estimation. The expression for this model is:


(1) $$y_{i}{\text{‏}}^{*}=\alpha +\beta x_{i}+\gamma s_{i}+\varepsilon_{i}$$

(2) $$y_{i}=\{\begin{array}{cc} 1, & y_{i}{\text{‏}}^{*}>0\\ 0 & y_{i}{\text{‏}}^{*}\leq 0 \end{array}$$

As shown in formula (1), $y_{i}{\text{‏}}^{*}$ represents the latent variable of the utilization of public health services for rural migrants. When *y*_i_*>0, *y*_*i*_ = 1, or *y*_*i*_ = 0_°_ *x*_*i*_ represents dialect culture, *s*_*i*_ are a series of control variables, mainly including gender, age, marital status, education level, self-assessment of health, migration time, range of migration, income level, distance from medical treatment, medical insurance, medical service level and city size. α, β, γ are parameters to be estimated, ε_*i*_ is a random disturbance term.

#### Instrument variable approach

The benchmark mentioned above is likely to have potential endogeneity issues for the following reasons. Firstly, the choice of the migration destination is based on costs and benefits. It results from self-selection to maximize income or utility rather than random selection. Directly estimating non-random samples will cause selection bias. Secondly, dialect culture is the carrier of regional culture. The impact of dialect culture on the utilization of public health services for rural migrants may be due to the influence of other cultural factors rather than the dialect culture itself, such as customs and living habits. These bilateral factors, which reflect the differences between the source and inflow areas of rural migrants, may not only be related to the dialect culture between the two places but also are important factors affecting the utilization of public health services for rural migrants. However, such factors are difficult to measure and characterize accurately and are often ignored in research models, leading to missing variables. Endogenous problems, as stated above, lead to inconsistent estimated coefficients.

To fix this issue, the typical solution is the instrument variable approach. This paper attempts to use the IVprobit model to carry out regression analysis. The specific model settings are as follows:


(3) $$y_{i}{\text{‏}}^{*}=a_{0}+a_{1}x_{i}+a_{j}s_{i}+u_{i}$$

(4) $$\begin{array}{lll} x_{i} & = & {\gamma_{1}z}_{i}+\gamma_{2}s_{i}+v_{i} \end{array}$$

(5) $$y_{i}=\{\begin{array}{cc} 1, & y_{i}^{*}>0\\ 0 & y_{i}^{*}\leq 0 \end{array}$$

From formula (3) to formula (5), *y*_*i*_ is an observable dummy variable, $y_{i}{\text{‏}}^{*}$ is an unobservable latent variable, *x*_*i*_ is the only endogenous variable in the model, *z*_*i*_ is an instrument variable, *u*_*i*_ and *v*_*i*_ is a random disturbance term. Suppose the disturbance term (*u*_*i*_, *v*_*i*_) obeys a two-dimensional normal distribution with an expected value of 0. That is:


(6) $$\left(\begin{array}{l} \text{‏}\\ u_{i}\\ \text{‏}\\ v_{i} \end{array}\right)\sim N\left[\left(\begin{array}{l} \text{‏}\\ 0\\ \text{‏}\\ 0 \end{array}\right),\left(\begin{array}{cc} 1\; & \rho \sigma_{v}\\ \rho \sigma_{v}\; & \sigma_{v}^{2} \end{array}\right)\right]$$

In the equation, the variance of *u*_*i*_ is normalized to 1, and ρ is the correlation coefficient of (*u*_*i*_, *v*_*i*_).

In addition, considering that the potential endogenous variable dialect culture is an ordered categorical variable, the IVprobit model ignores the ordered categorical variable attributes of dialect culture to a certain extent and cannot fully utilize the information, which results in the loss of estimation efficiency. So this paper further employs the Extended Probit (“Eprobit”) model in the Extended Regression Mode framework for re-estimation. For more details, please refer to the regression tool “eprobit” used in STATA. This method uses the Full Information Maximum Likelihood estimation method, which can more effectively deal with the endogenous variables, as in the case of ordinal categorical variables.

#### Mediation effect model

After analyzing the impact of dialect culture on rural migrants' public health service utilization, this paper further adopts Baron and Kenny's (55) mediation effect model, which takes information transmission, health habits, social capital, and cultural identity as mediating variables to examine the influence of dialect culture on rural migrants' public health services. In addition to formula (1) and formula (2), the specific path of health service utilization needs to construct the following measurement model:


(7) $$\begin{array}{lll} m_{i} & = & b_{0}+b_{1}x_{i}+b_{j}s_{i}+\varepsilon_{i} \end{array}$$

(8) $$y_{i}{\text{‏}}^{*}=c_{0}+c_{1}x_{i}+c_{2}m_{i}+c_{j}s_{i}+\varepsilon_{i}$$

(9) $$y_{i}=\{\begin{array}{cc} 1, & y_{i}{\text{‏}}^{*}>0\\ 0 & y_{i}{\text{‏}}^{*}\leq 0 \end{array}$$

Among them, $y_{i}{\text{‏}}^{*}$ represents the latent variable of the explained variable, when $y_{i}{\text{‏}}^{*}>0$, *y*_*i*_ = 1, otherwise, *y*_*i*_ = 0. *x*_*i*_ is the explanatory variable, and *m*_*i*_ is the mediator variable, including information transfer, healthy habits, social capital, and cultural identity. Based on the fact that Equations (1) and (2) have confirmed that dialect culture significantly hinders the public health services utilization for rural migrants, if both *b*_1_ and *c*_2_ are significant, there is an indirect effect. At this time, when *c*_1_ is not significant, there is a full mediation effect; when *c*_1_ is significant, there is a partial mediation effect.

In addition, considering that the mediation effect model proposed by Baron and Kenny is mainly aimed at the case where the explained variable is continuous, and to avoid potential bias in the estimation results, this paper adopts the KHB method proposed by Karlson et al. (56), which is suitable for the case where the explained variable is a discrete variable to test the robustness of the mediation effect.

## Empirical results

### Baseline regression results

Table 2 reports the benchmark regression results of the impact of dialect culture on rural migrants' utilization of public health services. To verify the robustness of the regression results, this paper adopts a stepwise regression method. Column (1) only controls the core explanatory variables, column (2) includes individual characteristics, column (3) includes urban characteristics, column (4) includes provincial dummy variables, and column (5) is the marginal effect of the estimated outcome of column (4). From the results in Table 2, it can be seen that whether only the core explanatory variables are controlled, individual characteristics, urban characteristics, or provincial dummy variables are added, the negative impact of dialect culture on the utilization of public health services for rural migrants is significant at the 1% level, indicating that the estimation results are robust. From the results in column (5), it can be seen that each time the dialect distance increases by one level, the possibility of rural migrants' utilization of public health services will decrease by 0.8%. The above results show that the more significant the dialect cultural difference, the lower the utilization of public health services for rural migrants. Thus, hypothesis 1 is preliminarily confirmed.

**Table 2:** Probit estimates of the effects of DCulture on PHSU of rural migrants.

**Variable**	**PHSU**				
	**(1)**	**(2)**	**(3)**	**(4)**	**(5)**
DCulture	−0.082^***^	−0.040^***^	−0.049^***^	−0.026^***^	−0.008^***^
	(0.003)	(0.004)	(0.004)	(0.005)	(0.001)
Gender		−0.074^***^	−0.079^***^	−0.084^***^	−0.026^***^
		(0.008)	(0.008)	(0.008)	(0.003)
Age		0.000	0.000	0.000	0.000
		(0.000)	(0.000)	(0.000)	(0.000)
Marriage		0.165^***^	0.167^***^	0.138^***^	0.043^***^
		(0.012)	(0.012)	(0.012)	(0.004)
Education		0.052^***^	0.064^***^	0.060^***^	0.019^***^
		(0.009)	(0.009)	(0.010)	(0.003)
Shealth		0.097^***^	0.105^***^	0.110^***^	0.034^***^
		(0.026)	(0.026)	(0.027)	(0.008)
Rtime		−0.003^***^	−0.003^***^	−0.001	−0.000
		(0.001)	(0.001)	(0.001)	(0.000)
FRange		0.117^***^	0.076^***^	0.017^**^	0.005^**^
		(0.007)	(0.007)	(0.008)	(0.002)
HIncome		−0.028^***^	−0.015^***^	0.002	0.001
		(0.006)	(0.006)	(0.006)	(0.002)
HDistance		−0.102^***^	−0.100^***^	−0.083^***^	−0.026^***^
		(0.009)	(0.009)	(0.010)	(0.003)
Medicare		0.215^***^	0.246^***^	0.240^***^	0.075^***^
		(0.010)	(0.010)	(0.011)	(0.003)
PHospital			−0.011	0.043^***^	0.014^***^
			(0.008)	(0.008)	(0.003)
SCity			−0.095^***^	0.041^***^	0.013^***^
			(0.005)	(0.008)	(0.002)
Constant	−0.356^***^	−0.574^***^	−0.415^***^	−1.565^***^	
	(0.009)	(0.057)	(0.058)	(0.073)	
Province effects	NO	NO	NO	YES	YES
Wald chi-squared	683.390^***^	2039.550^***^	2494.800^***^	10636.390^***^	10636.390^***^
Pseudo R^2^	0.005	0.015	0.018	0.081	
Observations	117,108	117,108	117,108	117,108	117,108

Robust standard errors in parentheses.

*** *p* < 0.01,

** *p* < 0.05. Marginal effect are reported in column (5) of this table.

Further, the control variables found that comparatively rural female migrants, rural male migrants had significantly lowers utilization of public health services. Moreover, marital status, education level, self-assessment of health, mobility range, and medical insurance significantly and positively influence the rural migrants' public health service utilization, meaning being married, having a high school education or above, physical health, closer mobility range, and having medical insurance of rural migrants have significantly higher utilization of public health services. The further the distance to seek medical treatment, the lower the utilization of public health services for rural migrants. The results of urban characteristics show that the areas' medical service level and city size significantly improve the utilization of public health services for rural migrants.

### Instrument variable result

Table 3 reports the estimated results of the IVprobit model. From the estimation results of the first stage of the IVprobit model in column (1), the instrument variable has a significant positive impact on dialect culture, which means that the instrument variable satisfies the correlation condition. From the estimation results of the second stage of the IVprobit model in column (2), the endogeneity test parameter of dialect culture is significant at the 1% level, indicating that dialect culture is indeed an endogenous variable, and the endogenous variable is just identified. The Anderson-Rubin test statistic also shows that the model does not have the problem of weak instrument variables, and the estimation results of the IVprobit model are more robust than the Probit model. From the estimation results, it is found that dialect and cultural differences significantly hinder the utilization of public health services for rural migrants; it can be seen that the negative impact of dialect culture on the utilization of public health services for rural migrants is robust, and research hypothesis 1 is further confirmed.

**Table 3:** IVprobit and Eprobit estimates of the effects of DCulture on PHSU of rural migrants.

**Variable**	**IVprobit**		**Eprobit**	
	**(1)**	**(2)**	**(3)**	**(4)**
	**DCulture**	**PHSU**	**DCulture**	**PHSU**
DCulture		−0.168^***^		−0.088^***^
		(0.039)		(0.017)
Instrument variable	0.199^***^		0.450^***^	
	(0.005)		(0.004)	
Wald test of exogeneity		13.100^***^		
AR weak Instrument variable test		18.180^***^		
Corr(e.Dculture, e.PHSU)				0.075^***^
				(0.021)
Constant	4.062^***^	−0.965^***^	2.339^***^	−1.395^***^
	(0.043)	(0.182)	(0.004)	(0.088)
Control variables	Yes	Yes	Yes	Yes
Province effects	Yes	Yes	Yes	Yes
Wald chi-squared		10,485.600^***^		10,389.880^***^
R^2^	0.526			
Observations	117,108	117,108	117,108	117,108

Robust standard errors in parentheses.

*** *p* < 0.01.

This paper further uses the Eprobit model, and according to the results in column (3), it can be seen that the instrument variables have a significant positive impact on the dialect culture, which also verifies that the instrument variables meet the correlation requirements. The main regression results in column (4) show that dialect culture significantly and negatively impacts the utilization of public health services for rural migrants, with a coefficient of −0.088. Compared with the coefficient in column (2), the coefficient becomes smaller, and the standard error decreases. It shows that the IVprobit model estimation has the problem of validity loss. The correlation test of residual items showed that the correlation between the regression model of endogenous variables and the primary regression model is significant, indicating that dialect culture is indeed an endogenous variable. After endogenous treatment, dialect culture significantly negatively impacts the utilization of public health services for rural migrants, which is highly consistent with the results of the IVprobit and probit models.

### Robustness check

Considering the endogenous problems such as sample selection and missing variables in the empirical model, this paper alleviates the problem by adding as many control variables as possible. It uses the instrument variable method to test the robustness of the analysis results. To further verify the reliability of the empirical results, this paper also uses four methods to conduct robustness tests, as shown in Table 4. First, the National Health and Family Planning Commission of China issued the Pilot Work Plan for Equalization of Basic Public Services for Health and Family Planning for Floating Population in 2013. Currently, there are 31 provincial-level administrative units in 44 cities with a high concentration of floating population in mainland China (see Table A1). Carry out pilot work on equalizing essential public services for health and family planning for the floating population. Rural migrants in the above 44 cities are likely to enjoy the same rights of public health services as residents. To better identify the impact of dialect culture on the utilization of public health services for rural migrants, this paper only retained samples from 44 key cities for robustness testing.

**Table 4:** Robustness test results.

**Variable**	**PHSU**				
	**(1)**	**(2)**	**(3)**	**(4)**	**(5)**
	**Key cities**	**Intra-city migration**	**Work and business**	**Under 60 years of age**	**Melogit**
DCulture	−0.014^*^	−0.046^***^	−0.023^***^	−0.026^***^	−0.016^*^
	(0.008)	(0.011)	(0.005)	(0.005)	(0.009)
Constant	−1.925^***^	−0.885^***^	−1.567^***^	−1.542^***^	−3.415^***^
	(0.188)	(0.131)	(0.084)	(0.076)	(1.157)
Control variables	Yes	Yes	Yes	Yes	Yes
Province effects	Yes	Yes	Yes	Yes	Yes
Pseudo R^2^	0.120	0.060	0.083	0.081	
var(_cons[city])					1.029^***^
					(0.102)
ICC					0.238^***^
					(0.018)
Observations	48,073	21,700	99,453	114,049	117,108

Robust standard errors in parentheses.

*** *p* < 0.01,

* *p* < 0.10.

The regression results are shown in column (1), and according to the results, it is found that the coefficient of dialect culture is significantly negative; indicating that even in 44 key cities, dialect culture still negatively impacts rural migrants' utilization of public health services. Secondly, in the city, urban and rural residents can enjoy public health services in the same city. Therefore, this paper only retained the samples that flow across counties within the city for robustness testing, and the regression results are shown in column (2). The results show that the coefficient of dialect culture is significantly negative; indicating that dialect culture still significantly reduces the probability of rural migrants' utilization of public health services even if they move to cities. Thirdly, only the samples of rural migrants who migrated for work and business were retained for robustness testing, and the regression results are shown in column (3). The results showed that dialect culture is significant, and the coefficient is negative, indicating that dialect culture still has a significant negative impact on the utilization of public health services for rural migrants. Fourthly, 3,059 rural migrants aged 60 and over in the sample used for the benchmark regression have reached the statutory retirement age but are still working. To further verify the robustness of the benchmark regression results, this paper only retained a sample of rural migrants under the age of 60 and re-estimated the impact of dialect culture on the utilization of public health services for rural migrants. The regression results in column (4) showed that dialect culture is negatively significant; indicating that dialect culture still significantly reduces the probability of rural migrants' utilization of public health services. Fifthly, Considering the regional effect of dialect culture, we re-estimated the relationship between dialect culture and rural migrants' public health service utilization using the melogit model, and the regression results are shown in column (5). The results show that the coefficient of dialect culture is significantly negative. Finally, it was considered that the use of ordered categorical variables to measure dialect culture differences between the origin and inflow of rural migrants may be biased because it implies that the distinction of the dialect culture differences between different situations are equal. Therefore, we examined the cases where rural migrants' source and inflow locations belonged to the same dialect piece, dialect area, and dialect region, respectively, as shown in columns (1), (2), and (3) of Table A2. The results show that same dialect mobility always exhibits higher utilization of public health services, regardless of the stratum. Furthermore, to make the results more refined, we compare only the cases where the dialects of the origin and inflow of rural migrants are exactly the same vs. completely different, and the results in column (4) of Table A2 show that same dialect area mobility significantly contributes to rural migrants' public health service utilization.

### Heterogeneity analysis

**Figure 4:**
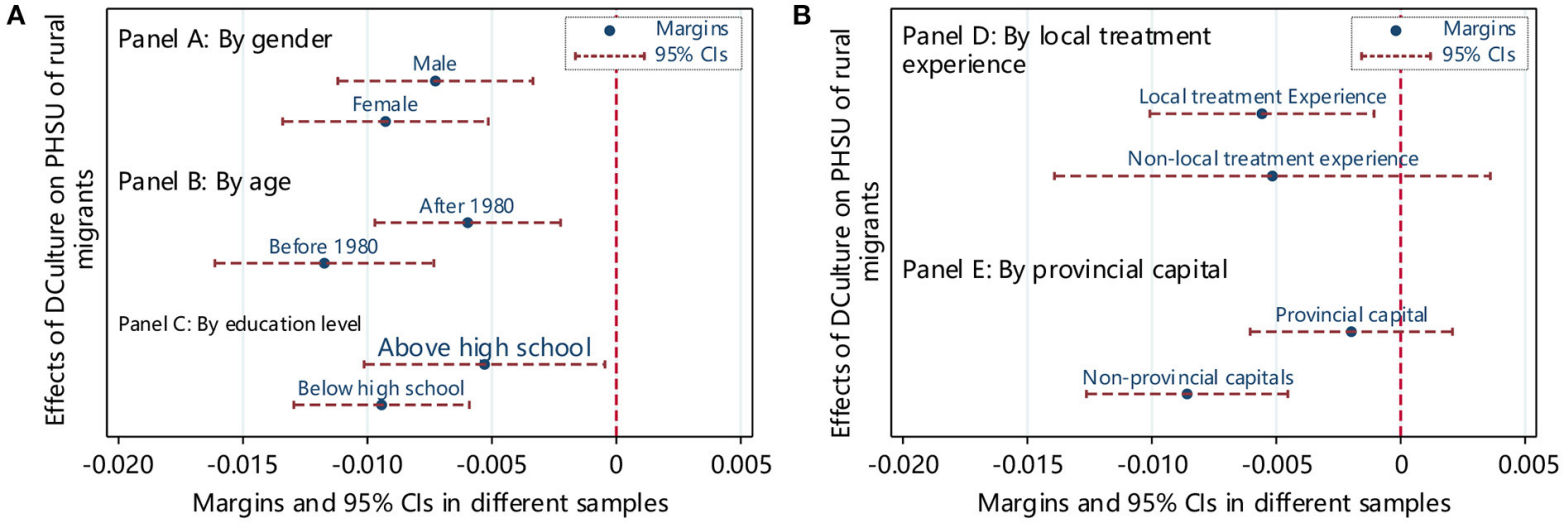
**(A,B)** The heterogeneous effect of DCulture on PHSU of rural migrants. The control variables introduced in the model fit were consistent with those in Table 2.

The previous analysis has confirmed that dialect culture hinders rural migrants' access to public health services but did not consider the heterogeneity. To obtain more detailed findings, the current study analyzed the heterogeneity from the perspective of migrating individuals and regions. The specific estimation results are shown in Figure 4.

Panels A, B, and C showed the grouped regression results based on gender, age, and education level. The results show that dialect culture significantly and negatively influences the utilization of public health services for rural migrants of different genders, ages, and educational levels. However, compared with males, rural migrants born after 1980 and with a high school education or above, dialect culture has a more significant impact on the utilization of public health services for women rural migrants born before 1980 and with below high school education. In addition, the group regression method to examine heterogeneity may be biased, so the current study employed the seemingly uncorrelated model (Suest) to test the differences between groups. The Suest test results showed that after grouping by gender, age, and education level, the coefficient differences between the groups are all significant at a 1% level, which further showed that the impact of dialect culture on the utilization of public health services for rural migrants is different based on gender and ages. And there are significant differences between groups with different educational levels. Panel D shows the difference based on having local medical treatment experience. The dialect cultural barriers of rural migrants without local medical treatment experience are insignificant.

In contrast, rural migrants with local medical treatment experience are more likely to reduce the utilization of public health services due to dialect and cultural differences. Panel E shows the difference between inflowing and non-provincial capital cities. The sample dialect culture of inflowing provincial capital cities is no longer significantly negative. Compared with provincial capital cities, dialect culture hinders public health services utilization for rural migrants who do not flow into provincial capital cities.

### Mediation analysis results

The results of the mediation effect analysis are shown in Table A3, columns (1)–(4) test the influence of dialect culture on the mediating variable, and columns (5)–(8) test the dialect culture and the four mediator variables included in the model on the utilization of public health services for rural migrants. Column (9) is listed as dialect culture, and the four intermediary variables are simultaneously included in the model for exploring the effects of rural migrants on the utilization of public health services. Columns (1)–(4) show that the regression coefficients of dialect culture on information transmission, healthy habits, social capital, and cultural identity of rural migrants are significantly negative; columns (5)–(8) show respectively that information transmission, health. The regression coefficients of habits, social capital and cultural identity on the use of public health services for rural migrants are all significantly positive; (9) under the control of dialect culture, information transmission, healthy habits, social capital and cultural identity, the coefficient of dialect culture is significantly negative. In contrast, the regression coefficients of information transmission, health habits, social capital, and cultural identity are all significantly positive. The above results show that information transmission, health habits, social capital, and cultural identity are all channels that influence the public health services of rural migrants by dialect culture.

In addition, the current study also estimated the mediating effects of information transfer, healthy habits, social capital, and cultural identity by employing the KHB method. The results of the KHB method are highly consistent with the estimation results obtained by the Baron and Kenny methods (see Table A4). According to the results, it is apparent that dialect culture indirectly hinders rural migrants' utilization of public health services through information transfer, healthy habits, social capital, and cultural identity, which further confirms the robustness of the results of the mediation effect. Thus Hypothesis 2 is also confirmed.

## Discussions

At present, the outburst of the COVID-19 pandemic has evolved into a global public health crisis (57, 58). Generally, it is regarded as a human, economic and social crisis. For preventing and controlling pandemics, the crucial role of the public health system cannot be ignored that upshot the urgent need to accelerate the improvement of the public health service system. Guo et al. (59) proposed to develop a sustainable social welfare system in China. In China, the quality of public health services accepted by rural migrants has become the “test gold stone” for the operation of the public health service system. However, as an essential carrier and symbol of regional culture, dialects, in addition to the function of communication, also represent the diverse cultural traditions and differentiated thinking habits of the language. In this study, we evaluated the impact of dialect culture on the utilization of rural migration public health services. According to the findings, it is apparent that the difference in dialect culture hinders the use of public health services for rural migrants. It infers that rural migrants have grown in a specific regional culture for a long time. After moving to cities, they are reluctant to adapt to new regional cultures. Moreover, rural migrants are more likely to be threatened by health threats due to high vocational risks and harsh living environments. It is easy to become blind spots for immunization, infectious disease prevention and control, and occupational health protection.

Furthermore, the cultural differences in dialect also led to information exchange between different groups, increasing information friction and restricting information exchange, and increasing the cost of collecting rural migration information (60), thereby hindering the spread of public health services policies. Therefore, it becomes difficult for rural migrants to accurately obtain information on local public health service projects, resulting in low use of their public health services. The findings corroborated the previous study by Wang et al. (61), who also showed that proficiency in Mandarin improves people's health and daily activities. Likewise, Strassmayr (62), in the case of developed countries, also exhibited the same findings and revealed that poor communication and cultural misunderstandings hinder public health services. Another finding is that comparatively rural male migrants born after 1980, and with high school or above, the dialect culture significantly hinders rural migrants born before 1980 and below high school. This means that women, the older and low-education rural migrants, are facing more cultural barriers and health inequality. The results are analogous to the recent studies that unveiled that rural women in developing countries confront cultural barriers (42, 63–65). The study proposes that government should promote and equalize essential health and public services primarily for the marginalized and deprived population following the study of Aziz et al. (66, 67). At the same time, rural migrants seeking medical treatment locally substantially affect their awareness of using public health services in the inflow place. Studies have found that the cultural hindrance of rural migrants with no local medical experience is insignificant. Rural migrants with local medical experience are more likely to reduce public health services due to cultural differences. This shows that when rural migrants encounter diseases and experience the public health services flowing place, the cultural obstacle of the dialect occurs. When rural migrants are healthy, dialect culture's impact is insignificant.

Moreover, the results of cultural hinders of rural migration dialects flowing into the provincial capital cities are no longer found to be significantly negative. Compared to non-provincial cities, migrants flow into the provincial capital cities obtains more public health services. The reason is that the provincial capital cities have more high-quality medical resources, and the service supply methods of grass-roots medical institutions make the rural migrants more accessible to health services. At the same time, as a political, economic, and cultural center in each province, the cultural diversity and inclusiveness of the provincial capital cities are more robust. The above findings provide a valuable reference for the provision of required public health service projects in government groups and regions. In addition, the current study finds that dialect culture indirectly hinders the public health services to rural migrants through information transmission, health habits, social capital, and cultural identity. The main reason behind this hindrance is the differences in Chinese dialects, voices, tone, and vocabulary that influence the information transmission and create challenges in using rural migration public health services. The results are parallel to the findings of Chen (68), who also revealed that language expressions directly affect the people's health in the long term (68). Likewise, Flores (69) also exhibited the same findings in the case of American medical services and stated that language barriers seriously hamper the efficiency of American medical services (69). Similarly, Zanchetta and Poureslami (70) also found that language, religion, and culture often cause communication barriers and reduce the efficiency of public health services obtained by migrant workers. Another reason behind the indirect influence of dialect culture on public health services is that dialect culture subtly affects the behavior, habits, and lifestyle of rural migrants. The cultural habits of rural migrants are deeply ingrained, creating many challenges in adapting to the living habits of new cities; consequently, it makes it difficult for migrants to use the public health services effectively. Abraído-Lanza et al. (52) scrutinized the impact of cultural adaptation on Latin migrants' health behavior, and risk factors became more unfavorable with the degree of cultural adaptation.

Last, it is also found that the differences between dialect cultures increase the social distance between rural migrants and residents, making rural migrants easily marginalized. Leigh (71) inferred that language differences reduce the trust of locals and migrants and the general trust of migrants. The language barrier affects the health level by reducing the ability to establish social networks for the elderly flowing population (50). In another study, Tanis and Postmes (72) believed that social group identity is an important feature affecting people's perception and judgment of other groups' credibility (72). Rural migrants grew up in different regional cultures, and the trust gap causes rural migrants to respond to the health management of public health services differently, thus causing a reduction in the utilization of public health services by rural migrants. Finally, due to the common existence of regional discrimination and prejudice, cultural symbols form the cornerstone of cultural identity. Through social classification, rural migrants have identified their groups and produced internal group preferences and external group prejudice, which hinders the use of public health services (73). Rural migrants have formed their cultural values while moving to cities. Suppose the cultural differences between the dialects flow into and outflow places persist. In that case, it makes it difficult for them to adapt new cultural identity, eventually reducing the utilization of rural migrants' public health services.

Based on the above discussion, it is apparent that the Chinese geographical environment, natural conditions, and historical inheritance exhibit different dialect cultures. Dialectical culture affects the rural migrants' cognition, interaction, and strategy choices of public health services. This study comprehensively evaluated the hindrances, heterogeneity, and mechanisms of dialect cultural hinders generated by the cultural flow on rural migration public health services. From the perspective of dialects, the invisible barriers to the non-equalization of rural migrant's public health services provide a factual basis for further understanding the equalization of rural migrants and public health services and provide evidence to promote the equalization of rural migrant's public health services. In the context of normalized prevention and control of COVID-19, the government should actively carry out cross-regional cultural exchange activities to balance cultural diversity and unity in development, thereby breaking down cultural barriers to the utilization of public health services for rural migrants.

## Conclusion and policy implications

Access to essential public health services is an essential aspect of social security. The quality of the services accepted by rural-to-urban migrants is a prominent reflection of the public health service system. However, it is also believed that the dialect culture influences the efficiency of public health policies. The current study aims to evaluate the impact of dialect culture on the utilization of rural public health services by rural migrants. By using the migrant data sourced from China Migrants Dynamic Survey (2017) and the dialect area information from the Chinese Dialect Dictionary (Revised Edition), the paper systematically examined the impact of dialect culture on the utilization of public health services for rural migrants. This study is enriching both cultural economics and health economics research literature in public goods provision. This paper finds that rural migrants with cross-cultural mobility face cultural barriers to public health equity, and dialect culture significantly hinders rural migrants' utilization of public health services, especially rural migrants who have local medical treatment experience and flow into non-provincial capital cities. In addition, the effect of dialect culture on the utilization of public health services by rural migrants not only directly but also indirectly hinders rural migrants' utilization of public health services through information transmission, healthy habits, social capital, and cultural identity.

In the normalization of prevention and control of COVID-19, the government should continuously optimize the public health service system, strengthen the publicity of essential public health service projects, and increase the enthusiasm and initiative of rural migrants to use public health services. Carrying out dynamic monitoring of rural migrants' health and open services for electronic health records, gradually eliminating isolated information of essential health for rural migrants. Relying on big data to achieve precise services and health supervision and improve the overall efficiency of the public health service systems. In addition, we also call on countries to attach great importance to the impact of invisible barriers caused by cultural differences on the efficiency of public health policies when carrying out public health actions and continuously improve the breadth and depth of exchanges and interactions between regional cultures to strengthen regional cultural identity and trust relationship—enhancing the tolerance and recognition of rural migrant groups in various regions and weakening the regional concept and social exclusion of rural migrants to eliminate social distance caused by cross-cultural mobility. Finally, promote immigrant integration.

Moreover, this study is not without limitations. First, the CMDS data used in this paper are cross-sectional data, not a longitudinal survey of rural migrants. Using a fixed-effect model to control the impact of individual differences that do not change over time is impossible. It may be difficult to thoroughly and clearly describe the current utilization of rural migrants' public health services and the dynamic changes in the structure. Secondly, due to the data source, the dialect culture calculation in this study only involves the Chinese dialect and does not consider the dialect data in ethnic minority areas. Chinese ethnic minority cultures have their characteristics with a long history, and there may be communication barriers and trust gaps so the future research should consider adding ethnic minority areas and dialects.

## Data availability statement

The original contributions presented in the study are included in the article/Supplementary material, further inquiries can be directed to the corresponding author.

## Author contributions

QZ wrote the main article. SX, NA, and YW revised and reviewed the article. JH supervised the article. All authors contributed to the article and approved the submitted version.

## Funding

This study was supported by the Humanities and Social Sciences Research Planning Fund of the Ministry of Education of China (Grant No. 20YJA790020), Tsinghua Rural Studies PhD Scholarship (Grant No. 202108), and Fundamental Research Funds for Humanities and Social Sciences of Nanjing Agricultural University (Grant No. SKYZ2020008).

## Conflict of interest

The authors declare that the research was conducted in the absence of any commercial or financial relationships that could be construed as a potential conflict of interest.

## Publisher's note

All claims expressed in this article are solely those of the authors and do not necessarily represent those of their affiliated organizations, or those of the publisher, the editors and the reviewers. Any product that may be evaluated in this article, or claim that may be made by its manufacturer, is not guaranteed or endorsed by the publisher.

## References

[1] Constitution of the World Health Organization. Available online at: https://www.who.int/about/governance/constitution (accessed June 20, 2022).

[2] Lucas R. On the mechanics of economic development. J Monet Econ. (1988) 22:3–42. 10.1016/0304-3932(88)90168-7

[3] United Nations,. Universal Declaration of Human Rights. (1948). Available online at: https://www.un.org/en/about-us/universal-declaration-of-humanrights (accessed June 22, 2022).

[4] Dickman SL Himmelstein DU Woolhandler S. Inequality and the healthcare system in the USA. Lancet. (2017) 389:1431–41. 10.1016/S0140-6736(17)30398-7 28402825

[5] Mostafavi F Piroozi B Mosquera P Majdzadeh R Moradi G. Assessing horizontal equity in health care utilization in Iran: a decomposition analysis. BMC Public Health. (2020) 20:914. 10.1186/s12889-020-09071-z 32532229 PMC7291751

[6] Meng X. Does a different household registration affect migrants' access to basic public health services in China? Int J Environ Res Public Health. (2019) 16:4615. 10.3390/ijerph16234615 31757111 PMC6926671

[7] Gu H Lin Y. Do you feel accepted? Perceived acceptance and its spatially varying determinants of migrant workers among Chinese cities. Cities. (2022) 125:103626. 10.1016/j.cities.2022.103626

[8] Hu X Sun M Tang S Lommel LL. Frequency of basic public health services utilization by married female migrants in China: associations of social support, discrimination and sociodemographic factors. BMC Womens Health. (2021) 21:344. 10.1186/s12905-021-01482-3 34583678 PMC8480003

[9] Zhou S Huang T Li A Wang Z. Does universal health insurance coverage reduce unmet healthcare needs in China? Evidence from the National Health Service Survey. Int J Equity Health. (2021) 20:43. 10.1186/s12939-021-01385-7 33478484 PMC7819183

[10] Lahana E Pappa E Niakas D. Do place of residence and ethnicity affect health services utilization? Evidence from Greece. Int J Equity Health. (2011) 10:16. 10.1186/1475-9276-10-16 21521512 PMC3107789

[11] Oliva-Moreno J Zozaya N López-Valcárcel BG. Opposite poles: a comparison between two Spanish regions in health-related quality of life, with implications for health policy. BMC Public Health. (2010) 10:576. 10.1186/1471-2458-10-576 20868523 PMC2955693

[12] Gu H Jie Y. Health service disparity, push-pull effect, and elderly migration in ageing China. Habitat Int. (2022) 125:102581. 10.1016/j.habitatint.2022.102581

[13] Al Shamsi H Almutairi AG Al Mashraf S Al KT. Implications of language barriers for healthcare: a systematic review. Oman Med J. (2020) 35:e122. 10.5001/omj.2020.40 32411417 PMC7201401

[14] Ali PA Watson R. Language barriers and their impact on provision of care to patients with limited English profciency: nurses' perspectives. J Clin Nurs. (2018) 27:e1152–60. 10.1111/jocn.14204 29193568

[15] Zhang D Jiang Z Xie Y Wu W Zhao Y Huang A. Linguistic barriers and healthcare in China: Chaoshan vs. Mandarin. BMC Health Serv Res. (2022) 22:1–10. 10.1186/s12913-022-07744-6 35317814 PMC8941784

[16] Shao S Wang M Jin G Zhao Y Lu X Du J. Analysis of health service utilization of migrants in Beijing using Anderson health service utilization mode. BMC Health Serv Res. (2018) 18:1–11. 10.1186/s12913-018-3271-y 29914464 PMC6006712

[17] Li J Shi L Liang H Ding G Xu L. Urban-rural disparities in health care utilization among Chinese adults from 1993 to 2011. BMC Health Serv Res. (2018) 18:1–9. 10.1186/s12913-018-2905-4 29426313 PMC5807772

[18] Xiao H Dai X Wagenaar BH Liu F Augusto O Guo Y. The impact of the COVID-19 pandemic on health services utilization in China: time-series analyses for 2016-2020. Lancet Regional Health-Western Pacific. (2021) 9:100122. 10.1016/j.lanwpc.2021.100122 34327438 PMC8315657

[19] Yang T Liu W. Does air pollution affect public health and health inequality? Empirical evidence from China. J Clean Prod. (2018) 203:43–52. 10.1016/j.jclepro.2018.08.242

[20] Zhang Z Hao Y Lu Z-N. Does environmental pollution affect labor supply? An empirical analysis based on 112 cities in China. J Clean Prod. (2018) 190:378–87. 10.1016/j.jclepro.2018.04.093

[21] Lagardea M Palmera N. The impact of user fees on health service utilization in low-and middle-income countries: how strong is the evidence? Bull World Health Organ. (2008) 86:839–48. 10.2471/BLT.07.049197 19030689 PMC2649541

[22] Ashraf N Berry J Shapiro J. Can higher prices stimulate product use? Evidence from a field experiment in Zambia. Am Econ Rev. (2010) 100:2383–413. 10.1257/aer.100.5.2383

[23] Hargreaves S Holmes AH Saxena S Feuvre P Chaudry P Friedland J. Charging systems for migrants in primary care: the experiences of family doctors in a high-migrant area of London. J Travel Med. (2008) 15:13–8. 10.1111/j.1708-8305.2007.00161.x 18217864

[24] Hiam L Mckee M. Making a fair contribution: is charging migrants for healthcare in line with NHS principles? J R Soc Med. (2016) 109:226–9. 10.1177/0141076816638657 27053358 PMC4908472

[25] Jacqueline B Martin M. Charging migrants for health care could compromise public health and increase costs for the NHS. J Public Health. (2016) 38:384–90. 10.1093/pubmed/fdv043 25904814

[26] Zhang F Shi X Zhou Y. The impact of health insurance on healthcare utilization by migrant workers in China. IJERPH. (2020) 17:1852. 10.3390/ijerph17061852 32178431 PMC7143864

[27] Lee DC Wang J Shi L Wu C Sun G. Health insurance coverage and access to care in China. BMC Health Serv Res. (2022) 22:1–9. 10.1186/s12913-022-07498-1 35114992 PMC8812221

[28] Hong Y Li X Stanton B Lin D Fang X Rong M. Too costly to be ill: healthcare access and health-seeking behaviours among rural-to-urban migrants in China. World Health Population. (2006) 2006:22–34. 10.12927/whp.2006.18280 18277099 PMC2249561

[29] Chen S Chen Y Feng Z Chen X Wang Z Zhu J. Barriers of effective health insurance coverage for rural-to-urban migrant workers in China: a systematic review and policy gap analysis. BMC Public Health. (2020) 20:408. 10.1186/s12889-020-8448-8 32228665 PMC7106835

[30] Li X Krumholz H Yip W Cheng K Maeseneer J Meng Q. Quality of primary health care in China: challenges and recommendations. Lancet. (2020) 395:1802–12. 10.1016/S0140-6736(20)30122-7 32505251 PMC7272159

[31] Loenen T Muijsenbergh M Hofmeester M Dowrick C Ginneken N Mechili E. Primary care for refugees and newly arrived migrants in Europe: a qualitative study on health needs, barriers and wishes. Eur J Public Health. (2018) 28:82–7. 10.1093/eurpub/ckx210 29240907

[32] Suphanchaimat R Kantamaturapoj K Putthasri W Prakongsai P. Challenges in the provision of healthcare services for migrants: a systematic review through providers' lens. BMC Health Serv Res. (2015) 15:390. 10.1186/s12913-015-1065-z 26380969 PMC4574510

[33] Chiu M Amartey A Wang X Kurdyak P. Ethnic differences in mental health status and service utilization: a population-based study in Ontario, Canada. Can J Psychiatry. (2018) 63:481–91. 10.1177/0706743717741061 29514512 PMC6099776

[34] Celik Y Hotchkiss DR. The socio-economic determinants of maternal health care utilization in Turkey. Soc Sci Med. (2000) 50:1797–806. 10.1016/S0277-9536(99)00418-9 10798333

[35] Ahmed S Creanga A Gillespie D. Economic status, education and empowerment: implications for maternal health service utilization in developing countries. PLoS ONE. (2010) 5:e11190. 10.1371/journal.pone.0011190 20585646 PMC2890410

[36] Tzogiou C Boes S Brunner B. What explains the inequalities in health care utilization between migrants and non-migrants in Switzerland? BMC Public Health. (2021) 21:1–15. 10.1186/s12889-021-10393-9 33736623 PMC7977586

[37] Kemppainen L Kemppainen T Skogberg N Kuusio H Koponen P. Migrants' use of health care in their country of origin: the role of social integration, discrimination and the parallel use of health care systems. Scand J Caring Sci. (2018) 32:698–706. 10.1111/scs.12499 28869656

[38] Elo I. Utilization of maternal healthcare services in Peru: the role of women's education. Health Trans Rev. (1992) 2:49–69. 10148665

[39] Wylie L Van Meyel R Harder H Sukhera J Luc C Ganjavi H. Assessing trauma in a transcultural context: challenges in mental health care with migrants and refugees. Public Health Rev. (2018) 39:1–19. 10.1186/s40985-018-0102-y 30151315 PMC6103972

[40] Aziz N Nisar Q Koondhar M Meo M Rong K. Analyzing the women's empowerment and food security nexus in rural areas of Azad Jammu and Kashmir, Pakistan: by giving consideration to sense of land entitlement and infrastructural facilities. Land Use Policy. (2020) 94:104529. 10.1016/j.landusepol.2020.104529

[41] Gu H Ling Y Shen T. How does rural homestead influence the hukou transfer intention of rural-urban migrants in China? Habitat Int. (2020) 105:102267. 10.1016/j.habitatint.2020.102267

[42] Wei W Sarker T Zukiewicz-Sobczak W Roy R Alam G Rabbany M. The influence of women's empowerment on poverty reduction in the rural areas of Bangladesh: focus on health, education and living standard. Int J Environ Res Public Health. (2021) 18:6909. 10.3390/ijerph18136909 34199117 PMC8293807

[43] Peled Y. Language barriers and epistemic injustice in healthcare settings. Bioethics. (2018) 32:360–7. 10.1111/bioe.12435 29741210

[44] Lara M Gamboa C Kahramanian M Morales L Hayes B. Acculturation and Latino health in the United States: a review of the literature and its sociopolitical context. Annu Rev Public Health. (2005) 26:367–97. 10.1146/annurev.publhealth.26.021304.144615 15760294 PMC5920562

[45] Khatri R Assefa Y. Access to health services among culturally and linguistically diverse populations in the Australian universal health care system: issues and challenges. BMC Public Health. (2022) 22:1–14. 10.1186/s12889-022-13256-z 35505307 PMC9063872

[46] Wang H Cheng Z Smythd R. Health outcomes, health inequality and Mandarin proficiency in urban China. China Econ Rev. (2019) 56:101305. 10.1016/j.chieco.2019.101305

[47] Salami B Salma J Hegadoren K. Access and utilization of mental health services for migrants and refugees: perspectives of immigrant service providers. Int J Ment Health Nurs. (2019) 28:152–61. 10.1111/inm.12512 29984880

[48] Lebrun L. Effects of length of stay and language proficiency on health care experiences among migrants in Canada and the United States. Soc Sci Med. (2012) 74:1062–72. 10.1016/j.socscimed.2011.11.031 22326103

[49] Rasi S. Impact of language barriers on access to healthcare services by immigrant patients: a systematic review. Asia Pacific J Health Manage. (2020) 2020:35–48. 10.24083/apjhm.v15i1.271

[50] Lu S Chen S Wang P. Language barriers and health status of elderly migrants: Micro-evidence from China. China Econ Rev. (2019) 54:94–112. 10.1016/j.chieco.2018.10.011

[51] Guiso L Sapienza P Zingales L. Cultural biases in economic exchange? Q J Econ. (2009) 124:1095–131. 10.1162/qjec.2009.124.3.1095

[52] Abraído-Lanza AF Chao MT Flórez KR. Do healthy behaviors decline with greater acculturation? Implications for the Latino mortality paradox. Social Sci Med. (2005) 61:1243–55. 10.1016/j.socscimed.2005.01.016 15970234 PMC3587355

[53] Liu Y Jiao Y Xu X. Promoting or preventing labor migration? Revisiting the role of language. China Econ Rev. (2020) 60:101407. 10.1016/j.chieco.2020.101407

[54] Kodagali V. Influence of regional and local topography on the distribution of polymetallic nodules in Central Indian Ocean Basin. Geo-Marine Lett. (1988) 8:173–8. 10.1007/BF02326094

[55] Baron RM Kenny DA. The moderator-mediator variable distinction in social psychological research: conceptual, strategic, and statistical considerations. J Pers Soc Psychol. (1986) 51:1173–82. 10.1037/0022-3514.51.6.1173 3806354

[56] Karlson KB Holm A Breen R. Comparing regression coefficients between same-sample nested models using logit and probit: a new method. Sociol Methodol. (2012) 42:286–313. 10.1177/0081175012444861

[57] Si R Yao Y Zhang X Lu Q Aziz N. Investigating the links between vaccination against COVID-19 and public attitudes toward protective countermeasures: implications for public health. Front Public Health. (2021) 9:702699. 10.3389/fpubh.2021.702699 34368065 PMC8333618

[58] Si R Lu Q Aziz N. Impact of COVID-19 on peoples' willingness to consume wild animals: empirical insights from China. One Health. (2021) 12:100240. 10.1016/j.onehlt.2021.100240 33898724 PMC8056415

[59] Guo B Xin X Qunhong W Xin Z Huaizhi C Sihai T. Inequality in the health services utilization in rural and urban China: a horizontal inequality analysis. Medicine. (2020) 99:e18625. 10.1097/MD.0000000000018625 31914043 PMC6959938

[60] Chiswick B Miller P. Occupational language requirements and the value of English in the US labor market. J Popul Econ. (2010) 23:353–72. 10.1007/s00148-008-0230-7

[61] Wang L Guruge S Montana G. Older migrants' access to primary health care in Canada: a scoping review. Can J Aging. (2019) 38:193–209. 10.1017/S0714980818000648 30777582

[62] Strassmayr C Matanov A Priebe S Barros H Canavan R Díaz-Olalla JM. Mental health care for irregular migrants in Europe: barriers and how they are overcome. BMC Public Health. (2012) 12:367. 10.1186/1471-2458-12-367 22607386 PMC3528475

[63] Aziz N Ren Y Rong K Zhou J. Women's empowerment in agriculture and household food insecurity: evidence from Azad Jammu and Kashmir (AJK), Pakistan. Land Use Policy. (2021) 102:105249. 10.1016/j.landusepol.2020.105249

[64] Lao X Zhao Z. Revisiting Hukou transfer intentions among floating population in chinese cities: spatial differences and multi-level determinants. SAGE Open. (2022) 12:21582440221097926. 10.1177/21582440221097926

[65] Aziz N Khan I Nadahrajan D He J. A mixed-method (quantitative and qualitative) approach to measure women's empowerment in agriculture: evidence from Azad Jammu and Kashmir, Pakistan. Commun Work Family. (2021) 2021:1–24. 10.1080/13668803.2021.2014783

[66] Aziz N He J Raza A Sui H Yue W. Elucidating the macroeconomic determinants of undernourishment in South Asian countries: building the framework for action. Front Public Health. (2021) 9:696789. 10.3389/fpubh.2021.696789 34458224 PMC8397478

[67] Aziz N He J Sarker T Sui H. Exploring the role of health expenditure and maternal mortality in South Asian countries: an approach towards shaping better health policy. Int J Environ Res Public Health. (2021) 18:11514. 10.3390/ijerph182111514 34770029 PMC8583359

[68] Chen M. The effect of language on economic behavior: evidence from savings rates, health behaviors, and retirement assets. Am Econ Rev. (2013) 103:690–731. 10.1257/aer.103.2.690 29524925

[69] Flores G. Language barriers to health care in the United States. N Engl J Med. (2006) 2006:229–31. 10.1056/NEJMp058316 16855260

[70] ZanchettaMSPoureslamiIM. Health literacy within the reality of immigrants' culture and language. Can J Public Health. (2006) 97:S28–33. 10.1007/BF0340537016805158

[71] Leigh A. Trust, inequality and ethnic heterogeneity. Econ Record. (2006) 82:268–80. 10.1111/j.1475-4932.2006.00339.x

[72] Tanis M Postmes TA. A social identity approach to trust: Interpersonal perception, group membership and trusting behaviour. Eur J Soc Psychol. (2005) 35:413–24. 10.1002/ejsp.25625222635

[73] Guiso L Sapienza P Zingales L. Does culture affect economic outcomes? J Econ Perspect. (2006) 20:23–48. 10.1257/jep.20.2.23

