# Supplementary material for: Dialect culture and the utilization of public health service by rural migrants: Insights from China
Source: Front Public Health. 2022 Nov 10;10:985343. doi: 10.3389/fpubh.2022.985343 (PMC9687394; doi:10.3389/fpubh.2022.985343)
Supplement: Supplementary file 1 [file Data_Sheet_1.docx]

**Appendix A**

**TABLE A1 List of key cities for the equalization of basic public health services for the floating population in China**

| **No.** | **Province** | **City** | **No.** | **Province** | **City** |
| --- | --- | --- | --- | --- | --- |
| 1 | Beijing | Chaoyang | 23 | Fujian | Xiamen |
| 2 |  | Fengtai | 24 |  | Quanzhou |
| 3 | Tianjin | Binhai | 25 | Jiangxi | Nanchang |
| 4 |  | Jinan | 26 | Shandong | Qingdao |
| 5 | Hebei | Shijiazhuang | 27 | Henan | Zhengzhou |
| 6 | Shanxi | Taiyuan | 28 | Hubei | Wuhan |
| 7 | Inner Mongolia | Baotou | 29 | Hunan | Changsha |
| 8 | Liaoning | Dalian | 30 | Guangdong | Shenzhen |
| 9 | Jilin | Changchun | 31 |  | Zhongshan |
| 10 | Heilongjiang | Harbin | 32 | Guangxi | Guilin |
| 11 | Shanghai | Minhang | 33 | Hainan | Sanya |
| 12 |  | Yangpu | 34 | Chongqing | Yubei |
| 13 |  | Songjiang | 35 | Sichuan | Chengdu |
| 14 |  | Baoshan | 36 | Guizhou | Guiyang |
| 15 | Jiangsu | Nanjing | 37 | Yunnan | Yuxi |
| 16 |  | Suzhou | 38 | Tibet | Lhasa |
| 17 |  | Wuxi | 39 | Shaanxi | Xi 'an |
| 18 | Zhejiang | Hangzhou | 40 |  | Xianyang |
| 19 |  | Ningbo | 41 | Gansu | Lanzhou |
| 20 |  | Jiaxing | 42 | Qinghai | Xining |
| 21 |  | Shaoxing | 43 | Ningxia | Yinchuan |
| 22 | Anhui | Hefei | 44 | Xinjiang | Karamay |

**TABLE A2 Effects of dialect consistency on public health service utilization among rural migrants**

| **Variable** | **PHSU** | | | | | | |
| --- | --- | --- | --- | --- | --- | --- | --- |
|  | **(1)** |  | **(2)** |  | **(3)** |  | **(4)** |
| DCulture |  |  |  |  |  |  |  |
|  |  |  |  |  |  |  |  |
| Same dialect piece | 0.061^***^ |  |  |  |  |  |  |
|  | (0.011) |  |  |  |  |  |  |
| Same dialect area |  |  | 0.063^***^ |  |  |  |  |
|  |  |  | (0.011) |  |  |  |  |
| Same dialect region |  |  |  |  | 0.031^***^ |  |  |
|  |  |  |  |  | (0.012) |  |  |
| Same dialect V.S. Different dialects |  |  |  |  |  |  | 0.054^***^ |
|  |  |  |  |  |  |  | (0.016) |
| Constant | -1.655^***^ |  | -1.653^***^ |  | -1.691^***^ |  | -1.690^***^ |
|  | (0.070) |  | (0.070) |  | (0.071) |  | (0.098) |
| Control variables | YES |  | YES |  | YES |  | YES |
| Province effects | YES |  | YES |  | YES |  | YES |
| Pseudo R^2^ | 0.081 |  | 0.081 |  | 0.081 |  | 0.081 |
| Observations | 117,108 |  | 117,108 |  | 117,108 |  | 75,628 |

Robust standard errors in parentheses. ***p < 0.01.

**TABLE A3 The effects of DCulture on PHSU of rural migrants: Mediating effect test results**

| **Variable** | **ITransfer** |  | **HHabits** |  | **SCapital** |  | **CIdentity** |  | **PHSU** | | | | | | | | |
| --- | --- | --- | --- | --- | --- | --- | --- | --- | --- | --- | --- | --- | --- | --- | --- | --- | --- |
|  | **(1)** |  | **(2)** |  | **(3)** |  | **(4)** |  | **(5)** |  | **(6)** |  | **(7)** |  | **(8)** |  | **(9)** |
| DCulture | -0.021^***^ |  | -0.049^***^ |  | -0.060^***^ |  | -0.022^***^ |  | -0.019^***^ |  | -0.025^***^ |  | -0.025^***^ |  | -0.026^***^ |  | -0.018^***^ |
|  | (0.004) |  | (0.005) |  | (0.004) |  | (0.004) |  | (0.005) |  | (0.005) |  | (0.005) |  | (0.005) |  | (0.005) |
| ITransfer |  |  |  |  |  |  |  |  | 1.412^***^ |  |  |  |  |  |  |  | 1.410^***^ |
|  |  |  |  |  |  |  |  |  | (0.011) |  |  |  |  |  |  |  | (0.011) |
| HHabits |  |  |  |  |  |  |  |  |  |  | 0.089^***^ |  |  |  |  |  | 0.035^***^ |
|  |  |  |  |  |  |  |  |  |  |  | (0.011) |  |  |  |  |  | (0.012) |
| SCapital |  |  |  |  |  |  |  |  |  |  |  |  | 0.063^***^ |  |  |  | 0.035^***^ |
|  |  |  |  |  |  |  |  |  |  |  |  |  | (0.008) |  |  |  | (0.009) |
| CIdentity |  |  |  |  |  |  |  |  |  |  |  |  |  |  | 0.060^***^ |  | 0.027^***^ |
|  |  |  |  |  |  |  |  |  |  |  |  |  |  |  | (0.008) |  | (0.009) |
| Constant | -0.723^***^ |  | 0.611^***^ |  | -0.688^***^ |  | -0.250^***^ |  | -2.209^***^ |  | -1.631^***^ |  | -1.581^***^ |  | -1.590^***^ |  | -2.253^***^ |
|  | (0.067) |  | (0.075) |  | (0.070) |  | (0.068) |  | (0.082) |  | (0.073) |  | (0.073) |  | (0.073) |  | (0.082) |
| Control variables | YES |  | YES |  | YES |  | YES |  | YES |  | YES |  | YES |  | YES |  | YES |
| Province effects | YES |  | YES |  | YES |  | YES |  | YES |  | YES |  | YES |  | YES |  | YES |
| Wald chi-squared | 6887.270^***^ |  | 5875.090^***^ |  | 8469.820^***^ |  | 3602.650^***^ |  | 20971.980^***^ |  | 10699.920^***^ |  | 10680.660^***^ |  | 10666.470^***^ |  | 20979.090^***^ |
| Pseudo R^2^ | 0.045 |  | 0.052 |  | 0.056 |  | 0.023 |  | 0.229 |  | 0.082 |  | 0.082 |  | 0.082 |  | 0.229 |
| Observations | 117,108 |  | 117,108 |  | 117,108 |  | 117,108 |  | 117,108 |  | 117,108 |  | 117,108 |  | 117,108 |  | 117,108 |

Robust standard errors in parentheses. ***p < 0.01.

**TABLE A4 The effects of DCulture on PHSU of rural migrants: Mediation effect estimation results based on KHB method**

| **Variable** | **PHSU** | | | | | | |
| --- | --- | --- | --- | --- | --- | --- | --- |
|  | **ITransfer** |  | **HHabits** |  | **SCapital** |  | **CIdentity** |
|  | **(1)** |  | **(2)** |  | **(3)** |  | **(4)** |
| Total effect | -0.030^***^ |  | -0.027^***^ |  | -0.026^***^ |  | -0.026^***^ |
|  | (0.005) |  | (0.005) |  | (0.005) |  | (0.005) |
| Direct effect | -0.019^***^ |  | -0.025^***^ |  | -0.025^***^ |  | -0.026^***^ |
|  | (0.005) |  | (0.005) |  | (0.005) |  | (0.005) |
| Indirect effect | -0.011^***^ |  | -0.001^***^ |  | -0.001^***^ |  | -0.001^***^ |
|  | (0.002) |  | (0.000) |  | (0.002) |  | (0.000) |
| Control variables | YES |  | YES |  | YES |  | YES |
| Province effects | YES |  | YES |  | YES |  | YES |
| Pseudo R^2^ | 0.230 |  | 0.080 |  | 0.080 |  | 0.080 |
| Observations | 117,108 |  | 117,108 |  | 117,108 |  | 117,108 |

Robust standard errors in parentheses. ***p < 0.01.
